# Data on the extent of sessile invertebrate fouling on the hulls of recreational boats in the western English Channel (north-east Atlantic), and patterns of boat maintenance and usage there

**DOI:** 10.1016/j.dib.2026.112803

**Published:** 2026-04-28

**Authors:** Christine Wood, Frédérique Viard, Anna Yunnie, Camille Bridge, John Bishop

**Affiliations:** a Marine Biological Association of the UK, Plymouth, UK; b ISEM, Univ Montpellier, CNRS, IRD, Montpellier, France; c Station Biologique de Roscoff, Sorbonne Université, CNRS, Roscoff, France; d Present address: PML Applications Ltd, Plymouth, UK

**Keywords:** Leisure craft, England, France, Non-native, Non-indigenous, Vector, Marine, Surveys

## Abstract

The movement of fouled leisure craft is recognized as a major vector for the spread of sessile marine non-indigenous species (NIS) along coastlines. Datasets are presented on the taxa found in the biofouling of the external wetted surfaces of recreational boats (sailing yachts and motor cruisers) in one coastal marina in Devon (SW England) and four coastal marinas in western Brittany (NW France). Visual inspections of the hull surface and ‘niche’ areas (rudder, propeller etc.) for sessile biota were carried out on 71 Devon and 50 Brittany boats immediately upon their removal from the water for maintenance, with field recording of suitable taxa and collection of specimens requiring laboratory identification. Twenty-four sessile NIS (23 invertebrates and one brown alga) were recorded. The distribution of fouling taxa between open-hull surfaces and niche areas was documented. Information was gathered on the recent maintenance regime of the boats studied, particularly with regard to hull cleaning and antifoul treatment, and on the usage patterns of the boats.

The fouling dataset characterizes typical loads of NIS carried by marine recreational boats and indicates which particular NIS are colonizing leisure craft, and at what frequency, in the western English Channel. This information can contribute to understanding patterns and mechanisms of the spread of marine NIS, particularly when combined with data on actual leisure-craft traffic volumes and patterns. Knowledge of typical patterns of cleaning and antifouling and their efficacy will help to shape biosecurity advice to marinas and boat owners.

## Graphical abstract

**Figure:**
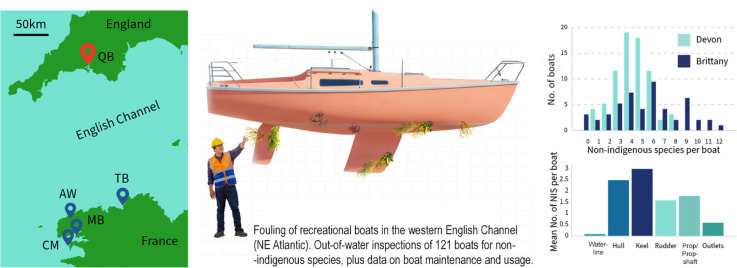

Specifications Table

**Table:** 

Subject	Biology
Specific subject area	The potential of leisure boats to spread marine non-indigenous species, and relevant patterns of maintenance and usage of the vessels.
Type of data	Table (.xlsx format), Document (PDF) Raw, plus summary data.
Data collection	Visual inspections of the hull surface and ‘niche’ areas for sessile biota were carried out on boats immediately upon their removal from the water for maintenance, with field recording of suitable taxa, collection of specimens requiring laboratory identification, and photography. Collected specimens were preserved in ethanol for laboratory identification using dissecting stereomicroscopes (Leica MZ6, MZ12.5). Interviews with boat owners were conducted in person at the time of removal of their boat from the water. The questions related to their boat maintenance regime and usage, and arose from a literature search [1,2], and from conversations with marina operators and boat owners.
Data source location	English data were collected from a single marina in Plymouth at 50.36494, -4.13115 (site code QB). French data were collected at four marinas in Brittany at 48.39214, -4.43325 (MB), 48.76997, -3.58664 (TB), 48.59859, -4.56133 (AW) and 48.27995, -4.59603 (CM), (Fig. 1).
Data accessibility	Repository name: Mendeley Data identification number: 10.17632/kxcdzzcvz8.1 Direct URL to data: https://data.mendeley.com/datasets/kxcdzzcvz8/1 Instructions for accessing these data: All datasets described in this paper are freely downloadable from the repository.
Related research article	None.

## 1 Value of the Data

• The movement of fouled leisure craft is recognized as a major vector for the spread of sessile non-indigenous species (NIS) along coastlines, but there is little information on the extent and taxonomic composition of leisure craft fouling by NIS in NW Europe, as compared to better-studied regions such as the Mediterranean [3], North America [4,5] and South Africa [6].
• The fouling data indicate which taxa are colonizing leisure craft and at what frequency. Most of the taxa were determined to species level, particularly non-indigenous taxa. This information might contribute to better understanding of traits or taxonomic groups that are particularly associated with the recreational boating vector.
• The patterns of usage observed could contribute to the identification of areas at most risk of transfer of NIS between sites. When combined with data on actual leisure-craft traffic volumes and patterns, for instance from the UK Automatic Identification System data [7], the information on levels of fouling could enable estimation of the amount of boat-mediated transport of particular NIS along different coastlines. This picture would inform practical management and policymaking concerning the arrival and spread of marine invasive species. Ten of the NIS in the English data set are included in the UK Marine Non-Indigenous Species Priority List for monitoring and surveillance [8].
• The inclusion here of data on taxa additional to those considered non-indigenous (here called ‘native’ taxa, including truly native species of the study area, as well as species that might be classified as cryptogenic, i.e. of uncertain native/non-indigenous status) is valuable in providing an approximate native:non-indigenous ratio, which would allow estimation of the non-indigenous presence amongst bulk estimates of total (unidentified) fouling.
• The data presented here exemplifies the option for taxonomically detailed documentation of fouling by out-of-water hull inspection immediately following removal of boats from the water. This approach has rarely been reported, although some of the extensive Mediterranean data presented by Ulman et al. (2019) [3] was gathered in this way. Underwater inspection of moored boats by remote cameras or divers is reported much more commonly in the literature, along with estimation of total fouling from the fouling visible from the surface (“dockside” recording) [1,2,9]. Part of our data is applicable to assessing the validity of using dockside observations to estimate total hull fouling.
• Information from the boat owner interviews indicating typical patterns of cleaning and antifouling could be used to shape biosecurity advice to marinas and boat owners. For instance, the data could inform environmental agencies designing outreach programmes and resources targeting yacht owners to reinforce biosecurity measures, such as the “Office Français de la Biodiversité” (French Office for Biodiversity; OFB) with the development of the “Econavigation” programme [10].

## 2 Background

• The data were generated between 2008 and 2011 as part of multi-partner investigations into the potential role of recreational boating and associated infrastructure (coastal marinas etc.) in the arrival, establishment and spread of marine NIS, particularly sessile fouling species. One key piece of information in this respect is the extent to which the external underwater surfaces of boats are colonized by NIS, which could then be transported from place to place, spreading species to new parts of the coast. The extent to which the taxonomic composition of boat fouling matches the composition of fouling populations on static structures in their home marinas is also relevant to understanding the mechanism of spread. Although the results of our parallel surveys of fouling populations in marinas have been published [e.g. 11], the boat-hull data have not, and we now want to make the boat data broadly available. There is no corresponding research article.

## 3 Data Description

**Fig. 1:**
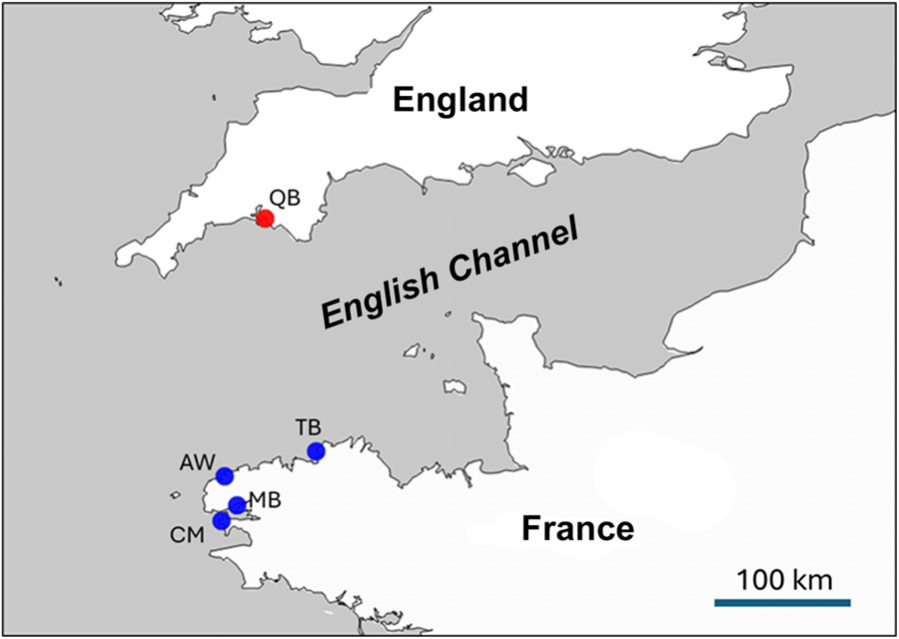
Map showing position of the marinas in the western English Channel where recreational boat hull inspections took place in Devon (England; red) and Brittany (France; blue).

Hull inspections were carried out from 2008 to 2011 in Devon, England, at a single marina, here referred to as QB. French inspections all took place in 2011 and involved four marinas in Brittany, France referred to as MB, TB, AW and CM (Fig. 1). To facilitate gaining access to the marinas it was agreed with the operators that the marina names would not be published.

### 3.1 Format of data in repository

Two multi-sheet Excel files (.xlsx) are provided, dealing respectively with the Devon and Brittany datasets. The two files provide similar information, with one additional sheet for the Brittany file (see below).

The Devon file has four sheets. Sheet 1 (GLOSSARY) indicates the contents of the subsequent three sheets and provides a glossary of their data fields and the abbreviations and codes used in each field. Sheet 2 (EN) gives presence/absence data for invertebrate and algal taxa on the hulls of 71 leisure craft surveyed between 2008 and 2011. For the 17 boats surveyed in 2011, Sheet 3 (EN HWO & KRP) divides the records for each boat into occurrences on the open-hull surface (including the waterline and outlets, labelled ‘HWO’) and those in the remaining ‘niche’ areas (base of keel, propeller including prop shaft, and rudder, ‘KPR’); the 2011 data is thus comparable to Sheet 3 in the Brittany file (see below). Sheet 4 (EN Questionnaire) gives basic information about the boats (type and size of boat, construction materials etc.), the date of the survey, and the results of questionnaire-based interviews concerning the maintenance and usage of their boats with those owners who were present and agreed to participate. Note that sheets 2 – 4 contain row and column subtotals, totals and summary values.

The Brittany file has five sheets. Sheet 1 (GLOSSARY) indicates the contents of the subsequent four sheets and provides a glossary of their fields and the abbreviations and codes used in each field. Sheet 2 (FR) gives presence/absence data for invertebrate and algal taxa recorded on the hulls of 50 leisure craft inspected in four Brittany marinas in 2011. Sheet 3 (FR HWO & KRP) splits the records for each boat into occurrences on the open hull surface (including the waterline and outlets, labelled ‘HWO’) and those in the remaining ‘niche’ areas (base of keel, propeller including prop shaft, and rudder, ‘KPR’). Sheet 4 (FR Boat sections) has the occurrences further divided into six areas: waterline, hull, base of keel, rudder, prop plus prop shaft, and outlets. Information at this level of detail is not matched in the Devon data set. Sheet 5 (FR Questionnaire) gives basic information about the boats (type and size of the boat, construction materials etc.), the date of the survey, and the results of questionnaire-based interviews with the owners concerning the maintenance and usage of all the surveyed boats. Sheets 2 – 5 contain row and column subtotals, totals and summary values.

The English questionnaire and a translated version of the French questionnaire are provided in PDF format in the repository and as supplementary material here.

### 3.2 Results **–** species data

**Fig. 2:**
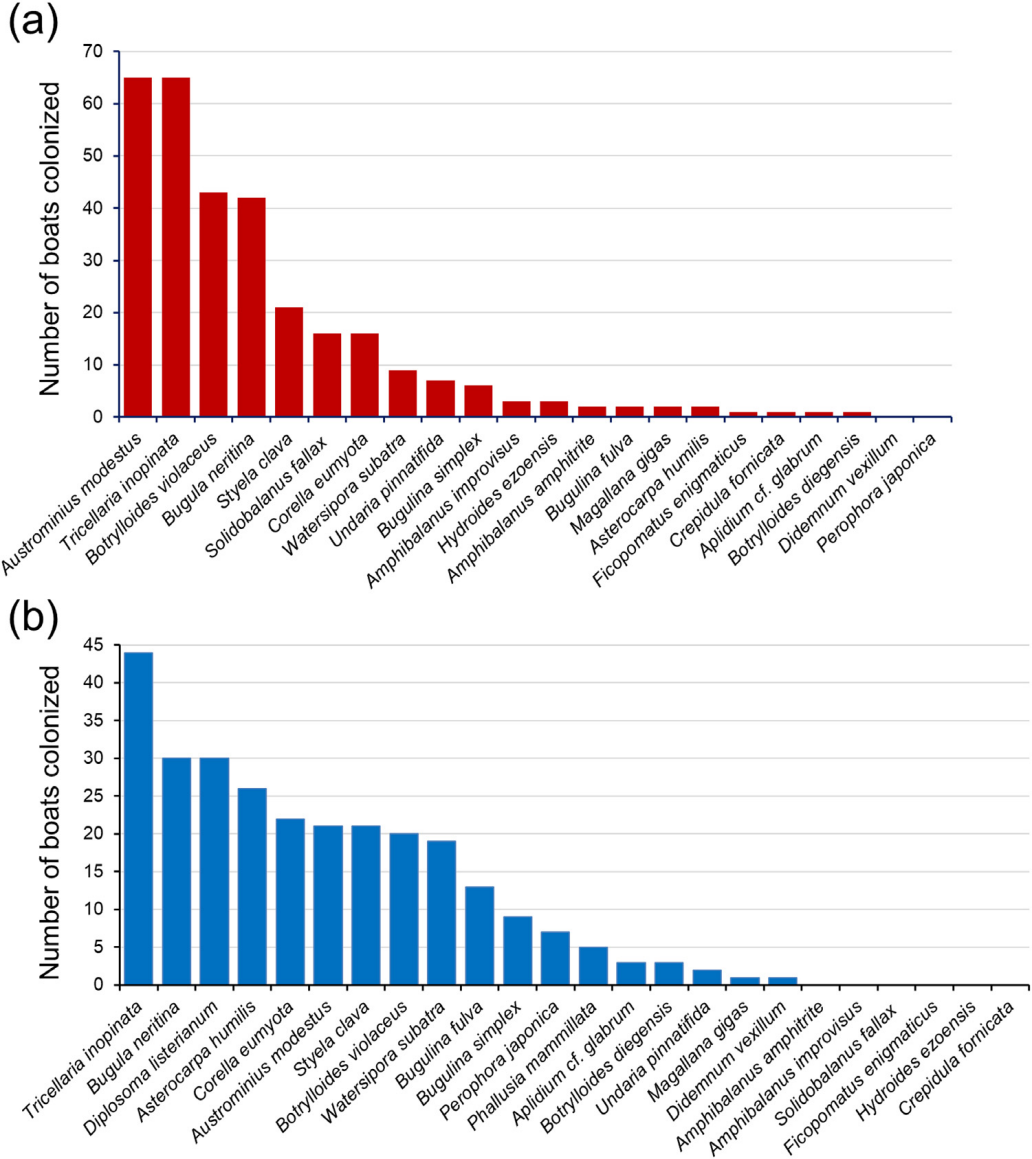
Ranked bar charts of the number of boats colonized by each NIS in (a) Devon (n = 71 boats) and (b) Brittany (n = 50 boats).

**Fig. 3:**
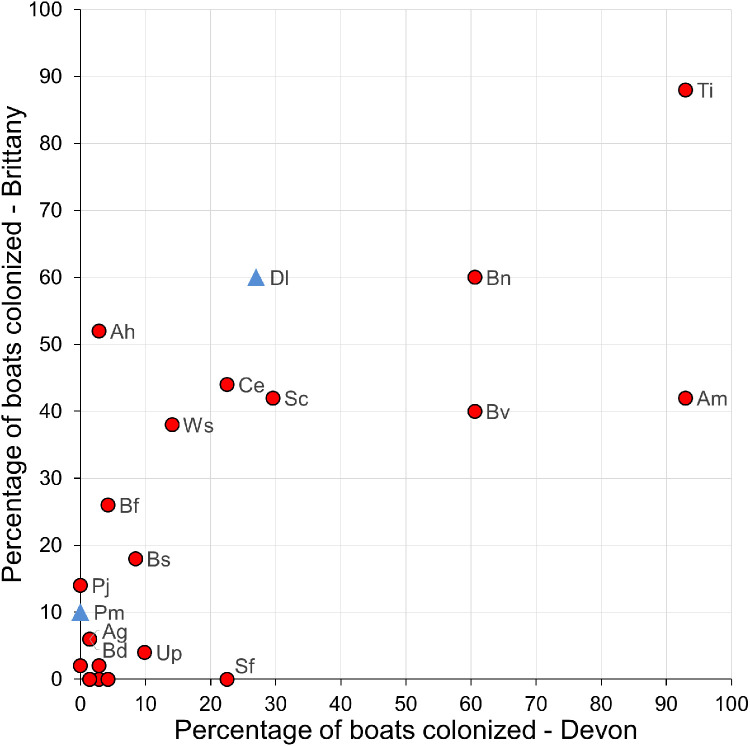
Bivariate scatter plot of the percentage of boats in Devon and Brittany colonized by each NIS. Species with blue symbols are regarded as non-native in France but native in Great Britain, red symbols are non-native in both. The unlabelled species in the cluster very close to the origin are *Aa, Ai, Cf, Dv, Fe, He* and *Mg*. The two-letter labels are the initials of each species’ genus and species names (see Table 1).

Twenty species recognized as non-indigenous in Great Britain were recorded on the Devon boats and 18 French NIS on the Brittany boats (Fig. 2, Fig. 3; see also Table 1). The upright-growing bryozoan *Tricellaria inopinata* was the most frequently occurring species on Brittany boats and jointly the most frequent, along with the barnacle *Austrominius modestus*, on the Devon boats; *T. inopinata* occurred on c. 90% of boats in both data sets. A second upright bryozoan, *Bugula neritin*a, was found on c. 60% of boats in both data sets, while two colonial ascidians, *Diplosoma listerianum* and *Botrylloides violaceus* occurred on c. 60% of Brittany and Devon boats respectively. The unitary ascidian *Asterocarpa humilis* was notably more frequent in Brittany (present on 26 out of 50 boats) than in Devon (2 out of 71 boats). This observation matches the rapid infilling of sites by *A. humilis* seen in SW English marinas between 2010 and 2013, while Brittany marinas were already all colonized by 2010 [11].

**Table 1:** The presence of 22 species non-indigenous in both GB and France and the two additional species regarded as non-indigenous only in France (asterisks*) recorded in the data set. All are invertebrate animals except for *Undaria pinnatifida* (a brown alga). The two-letter abbreviations used in Fig. 3 are shown. Presence on Devon and Brittany boats is indicated with a Y.

Non-indigenous Species	Fig. 3 label	Devon	Brittany
*Amphibalanus amphitrite*	Aa	Y	
*Amphibalanus improvisus*	Ai	Y	
*Aplidium* cf. *glabrum*	Ag	Y	Y
*Asterocarpa humilis*	Ah	Y	Y
*Austrominius modestus*	Am	Y	Y
*Botrylloides diegensis*	Bd	Y	Y
*Botrylloides violaceus*	Bv	Y	Y
*Bugula neritina*	Bn	Y	Y
*Bugulina fulva*	Bf	Y	Y
*Bugulina simplex*	Bs	Y	Y
*Corella eumyota*	Ce	Y	Y
*Crepidula fornicata*	Cf	Y	
*Didemnum vexillum*	Dv		Y
Diplosoma listerianum*	Dl*	Y*	Y*
*Ficopomatus enigmaticus*	Fe	Y	
*Hydroides ezoensis*	He	Y	
*Magallana gigas*	Mg	Y	Y
*Perophora japonica*	Pj		Y
Phallusia mammillata*	Pm *		Y*
*Solidobalanus fallax*	Sf	Y	
*Styela clava*	Sc	Y	Y
*Tricellaria inopinata*	Ti	Y	Y
*Undaria pinnatifida*	Up	Y	Y
*Watersipora subatra*	Ws	Y	Y

**Fig. 4:**
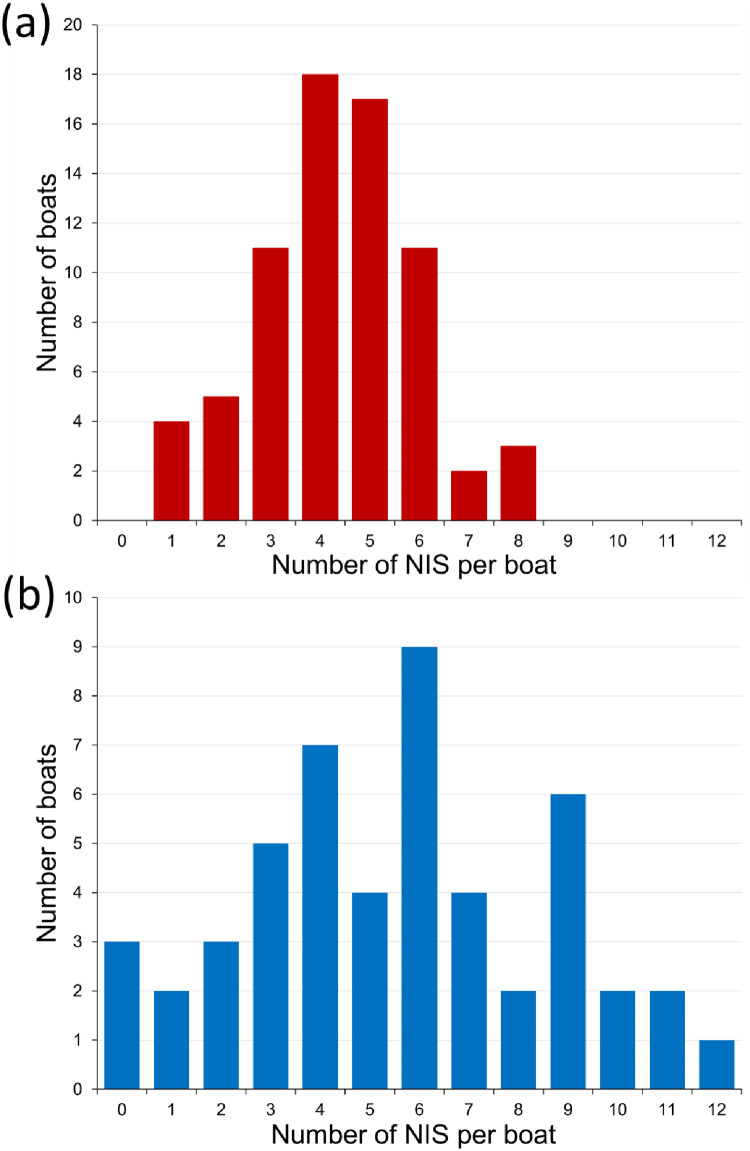
Frequency plots of number of NIS recorded per boat in (a) Devon (n = 71; mean 4.34, S.D. 1.63 NIS per boat) and (b) Brittany (n = 50; mean 5.54, S.D. 3.05 NIS per boat). (Brittany data include *Diplosoma listerianum* and *Phallusia mammillata*, not considered non-native in Great Britain).

Devon boats carried an average of 4.32 NIS, with a range of one to eight NIS per boat (Fig. 4a), while Brittany boats averaged 5.54 NIS per boat, ranging from zero to 12 NIS per boat (Fig. 4b). Thus, none of the Devon boats were free of NIS while 6% of Brittany boats lacked NIS. The Brittany NIS data quoted above include *D. listerianum* and *Phallusia mammillata*, species considered native in Great Britain. (For a more direct comparison using only species categorised as NIS in both regions the Brittany mean becomes 4.84 per boat, rather than 5.54, compared to 4.32 in Devon.) Comparing the three French ports with more than one boat (i.e. excluding site CM), and again including *D. listerianum* and *P. mammillata*, the largest value was observed at TB with 6.5 NIS on average per boat (n = 18), as compared to 3.9 in AW (n = 10) and 5.4 in MB (n = 21); the differences were not significant (Kruskal-Wallis test, *P* = 0.16).

On the Devon boats, the number of NIS recorded on the niche areas (KRP; mean 4.47) was significantly greater than on the open-hull areas (HWO; mean 2.35) (Paired T-test, *P* < 0.001). The same was true of the total count of non-indigenous and native taxa for the two areas: KRP mean 14.35, HWO mean 7.94; *P* = 0.001.

The same distinction between niche and open-hull areas was observed on the Brittany boats: for NIS, KRP mean 3.94 species, HWO mean 2.42; *P* < 0.001; for all taxa, KRP mean 10.68, HWO mean 7.64; *P* = 0.005.

**Fig. 5:**
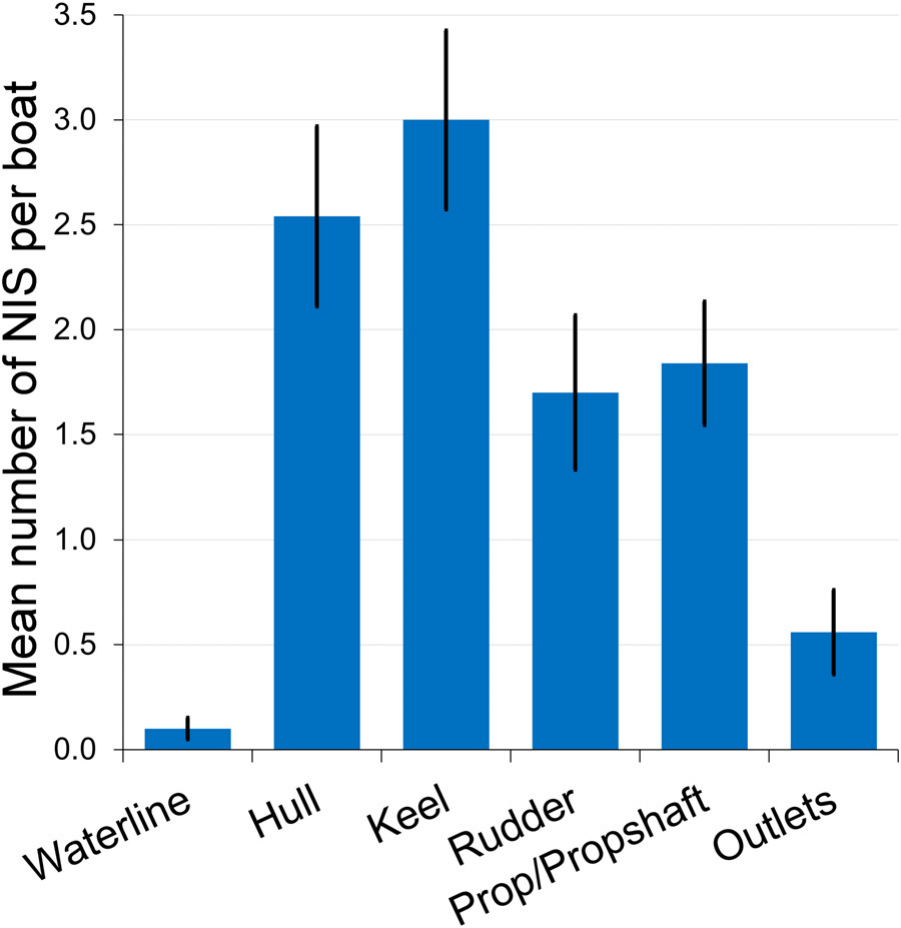
The mean number (± S.E.) of NIS per boat recorded in six different regions of the underside of leisure craft (n = 50) in Brittany marinas.

The mean numbers of NIS detected on Brittany boats in various niche areas and on the main hull surface are displayed in Fig. 5. It is apparent that fouling along the waterline involves very few species while niche features such as the keel, rudder and propeller carry relatively high numbers of different NIS despite their limited areal extent*.*

Comparison of the boat-hull data with data from repeated rapid assessment surveys (RASs) of marinas QB, MB, TB, and AW in 2010 and 2013, reported by Bishop et al. (2015) [11], is included as a table in the supplementary material. Six taxa occurred in the boat data and in both repeated RASs across all four marinas: *Corella eumyota, Styela clava, Botrylloides* spp., *B. neritina, T. inopinata* and *Austrominius modestus*. In Brittany, *Didemnum vexillum* was recorded in the three marinas in all six RASs but only in a single marina on boats, and was not recorded at all in QB (Devon). Unlike the Brittany sites, QB showed an increase in NIS between the two RASs, reflecting ongoing infilling of vacant sites on the south coast of England [11]. Two of the additions at QB, *A. humilis* and *Watersipora subatra*, were recorded on the boats first, before being recorded for the first time on the marina pontoons at QB during the 2013 RAS. (Although *Crepidula fornicata* shows the same pattern of occurrence at QB in the supplementary table as *A. humilis* and *W. subatra*, it is a much older introduction which is long established in the region but is primarily a seabed, rather than fouling, species.)

**Fig. 6:**
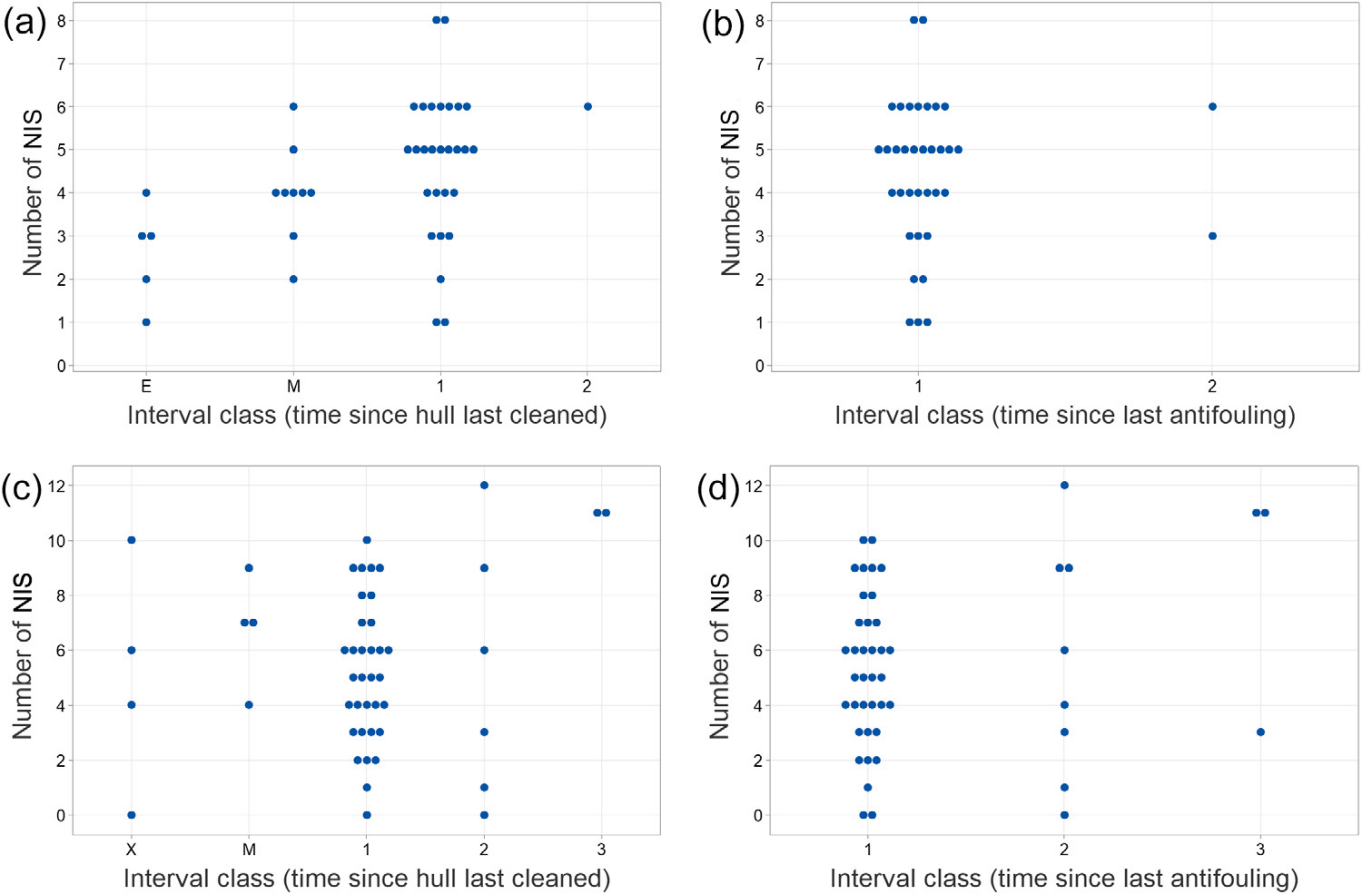
The number of NIS recorded on Devon (a, b) and Brittany (c, d) leisure craft at different intervals since their underwater surfaces were last cleaned (left; a, c) or antifouled (right; b, d). Interval classes 1, 2 and 3 are approximately 1, 2 and 3 years respectively, and letter codes are for lesser intervals, but see Table 2 for full explanations of the classes. Number of boats scored: 6a, 43; 6b, 36; 6c, 49; 6d, 47.

The numbers of NIS recorded on individual Devon and Brittany boats are displayed in Fig. 6a–d in relation to the length of the intervening period between the time of survey and the time each boat was last cleaned or cleaned and antifouled. The various intervals were referred to a number of ‘interval classes’ according to a scheme explained more fully in Table 2; classes 1, 2 and 3 equate approximately to those numbers in years. It is evident that most boats in both data sets receive antifouling (invariably preceded by a preparatory clean) once a year. The strong preponderance of data for the one-year class reduces the ability to link levels of fouling to the time elapsed since the last cleaning or antifouling. Nevertheless, the diagram for fouling on Devon boats in relation to time since most recent hull cleaning (Fig. 6a) does suggest an increase in fouling diversity over time.

**Table 2:** Last clean and last antifoul interval classes derived from date of last clean or antifoul respectively. Sailing season presumed to be April **–** October on both coasts. English surveys undertaken September **–** March in 2008 **–** 2011. French surveys undertaken March **–** May 2011.

Last clean /antifoul interval class		Time period (most recent **first**)
**Devon**	E	End of last season (October – November)
	M	Intermediate in the last season (May – September)
	1	Start of last season (October – April)
	2	Start of season two years ago (October – April)
	3	Start of season three years ago (October – April)
		
**Brittany**	X	In current season (March – April 2011)
	M	Intermediate in the last season (June – October 2010)
	1	Start of last season (March – May 2010)
	2	Start of season two years ago (March – May 2009)
	3	Start of season three years ago (March – May 2008)
	A	Other

The effect of the longest continuous period of mooring in the previous 12 months on the number of NIS recorded was investigated. For the Devon boats the periods of mooring were divided into ≤1 month, 1 – 3 months and >3 months; no significant overall effect was apparent (Kruskal-Wallis test, *P* = 0.716).

**Fig. 7:**
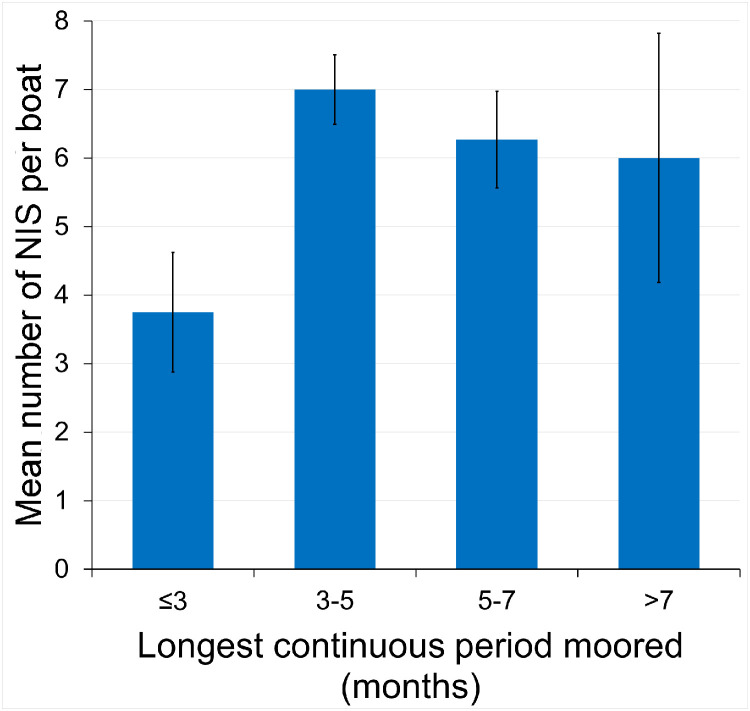
Mean number (± S.E) of NIS per boat for 50 leisure craft in Brittany marinas classified according to their longest period of inactivity in the previous 12 months.

For the Brittany boats (n = 44), the longest continuous periods of mooring in the previous 12 months adopted were ≤3 months, 3 – 5 months (including 5.0), 5 – 7 months (including 7.0), and >7 months (Fig. 7). A significant overall effect was apparent (Kruskal-Wallis test, *P* = 0.046). Of the six Mann-Whitney tests comparing the four different periods pairwise, only two comparisons, both involving the low NIS counts for the shortest interval, were significant: <3 vs. 3 – 5 months (P = 0.008) and <3 v. 5 – 7 months (*P* = 0.030). In this dataset colonization by NIS appeared to be restricted in the absence of prolonged periods of inactivity.

### 3.3 Results – boat characteristics, maintenance and usage

On both sides of the Channel, the majority of vessels inspected were yachts (sailing boats) (Devon 81.4%, Brittany 62.0%) and the great majority of all boats had fibreglass/glass-reinforced plastic hulls (Devon 98.5%, Brittany 92.0%).

**Fig. 8:**
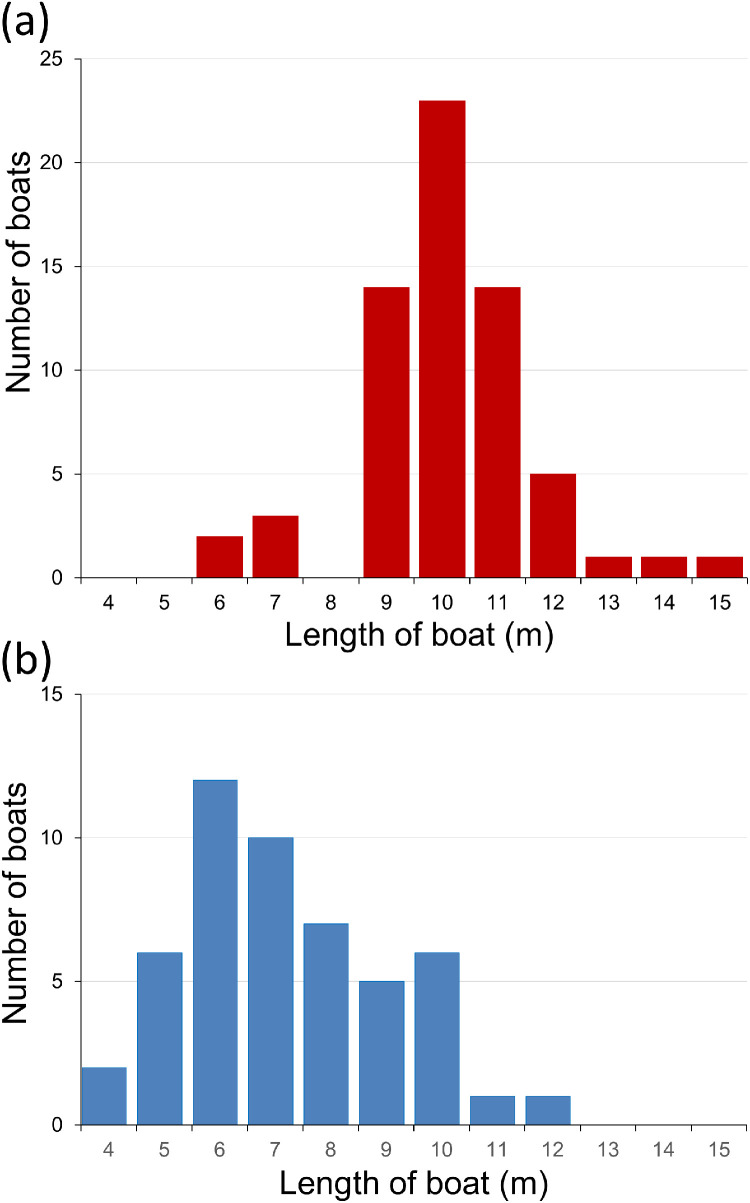
Frequency plots of lengths of the boats surveyed. (a) Devon (64 boats measured, mean 10.58 m, S.D. 1.58 m); (b) Brittany (50 boats measured, mean 7.82 m, S.D. 1.93 m).

The boats surveyed in Devon had a greater median length than those in Brittany (Mann-Whitney U test, *P* < 0.001) (Fig. 8). Devon and Brittany boats ranged from 6.1 to 15.2 m and from 4.6 to 12.4 m in length respectively. The yachts inspected within a region were longer overall than the motor boats in the same region in both Devon (Mann-Whitney U test, *P* = 0.009) and Brittany (Mann-Whitney U test, *P* < 0.001).

In Brittany, the majority of respondents (88%) cleaned and antifouled their own boats, a similar pattern being suggested by the small number of Devon respondents. Seventy-five percent of Devon respondents used a roller to apply antifouling paint, with a further 15% using a combination of roller and brush. The vast majority (93%) of Brittany respondents used a roller with only two (5%) using a combination of roller and brush and a single respondent (2%) a spray-gun. Ablative antifouling coatings were the most popular type used in Devon (64%), followed by hard antifoul (18%) and mixed-matrix formulations (10%). However, in Brittany hard antifouls were predominant (73%) followed by ablative and mixed-matrix types (both 12%).

In Devon 70% of boats were berthed at the marina where the inspections were conducted and a further 11% were berthed in a second marina immediately adjacent; the remainder were one or two boats visiting from each of seven other south-west England marinas from as far west as Falmouth (Cornwall) and as far east as Poole (Dorset). In Brittany all boats were inspected in their home marinas with the exception of three craft from nearby marinas that were inspected at MB.

Information on places visited was obtained for 46 Devon boats. Twenty-three of these (50%) stayed within the South Coast of England, from Falmouth (Cornwall) in the west to the Solent (Hampshire) in the east. Six boats visited the Isles of Scilly and one went as far north as Scotland. Twenty-four cross-Channel visits were logged amongst 19 different English vessels: 14 visits to the Channel Isles, nine to France (eight to Brittany, one to Normandy) and one to The Netherlands.

The 50 French vessels, all berthed in Brittany, mostly went to the southern or northern coasts of their home administrative region. Two French vessels visited the Channel Islands, and five crossed the English Channel, four to the English South Coast and one to the Thames.

## 4 Experimental Design, Materials and Methods

Boats were surveyed at a single English marina in Devon (SW England) and four different French marinas in NW Brittany (NW France). These regions are near the western end of the English Channel, on opposite sides of the Channel at very similar longitudes (Fig. 1).

The actual collecting of biological data was comparable between England and France; one surveyor participated in both sets of surveys, ensuring correspondence of the practical survey process and taxonomic understanding. For the fouling taxa considered, the respective lists of established NIS are very similar between Great Britain [12] and France [13]. However, according to these lists the ascidians *D. listerianum* and *P. mammillata* are considered non-indigenous on the French English Channel coast [13] (Massé et al., their Table S1) but native in Great Britain. Note that the column NIS occurrence totals in sheet FR of the Brittany Excel file do not include *D. listerianum* or *P. mammillata*.

In both England and France, hull fouling was investigated on land soon after the removal of leisure craft from the water for maintenance. In England this involved the scheduled craning of a boat out of the water by marina personnel up onto a ramp above the water level where it was temporarily propped upright. The researchers were allowed access to the newly exposed underside of the boat for approximately three minutes before marina personnel started removing any fouling with a high-pressure water hose (followed by transfer of the boat onto hard standing). During the period of access all wetted regions of the boat were inspected by three researchers, species noted, photographs taken, and specimens collected for subsequent identification. In addition to non-indigenous species, other algal and sessile invertebrate taxa (i.e. natives and those considered cryptogenic) were recorded. Plastic scrapers were used for collecting specimens to avoid damaging the boat’s surfaces. Initial identifications were then made at the marina with the specimens spread out in a tray of seawater, with examples preserved as necessary in 70% ethanol for lab identification. In 2011 only, separate records were made of A) fouling on general hull surfaces normally receiving conventional antifouling and B) so-called ‘niche’ areas, difficult or inappropriate to treat with regular antifoul paints: the base of the keel, the propeller plus propeller shaft, and the rudder; in earlier years this distinction was not made during recording.

In France, a proportion of boats was inspected in the same circumstances as in Devon, soon after the boat was lifted or hauled out of the water by marina staff. Other boats were grounded on a ramp at high tide by the owner to become exposed as the tide fell. In either case a single researcher spent 10 min examining the exposed underside of each boat, separately recording fouling on the hull, propeller plus shaft, rudder, base of keel and openings of inlet/outlet pipes.

Laboratory identification of specimens was based on morphology using dissecting stereomicroscopes and standard identification literature. DNA barcoding was not undertaken. Taxonomic queries were discussed and agreed between the English and French participants. External taxonomic advice was sought as necessary. Taxonomic names used here have been updated according to WoRMS [14].

In the English marina the boat’s owner was interviewed by one of the three researchers before or after the period of access to the boat to record fouling. However, boats were often lifted by the marina staff without the owner being present, in which case the owner questionnaire could not be completed.

In France every boat’s owner was present and was interviewed by a second researcher while the hull inspection was going on.

The questionnaire sheet used in England and a translated version of the French questionnaire have been deposited in the repository and are provided in the supplementary material for this article.

## Limitations

Only sessile species were recorded. Our main focus was on NIS; native taxa were often recorded with less taxonomic resolution. Identification of algae was very limited.

Five Devon boats were surveyed twice: D32/D63; D38/D64; D39/D66; D41/D65 and D43/D67. Each of these boats was cleaned and re-antifouled between the surveys, so the fouling scored represented two different colonization events. For some potential analyses the duplicated information may need to be discarded. One boat was surveyed in both Devon and Brittany: D38/D64 = B3.

Some factors reduced the comparability of the French and English data sets. Seasonal and year-related differences are possible due to the different survey times. *D. vexillum* and *P. mammillata* are categorized as non-indigenous in France but native in GB, a difference of scientific opinion unlikely to reflect a genuine biogeographical distinction. To allow more direct comparison between Devon and Brittany, these were not included in NIS totals in the Brittany spreadsheet. In further analyses representing a purely French NIS perspective, they should be included. The English boats were generally larger than the French (Fig. 8). The absence of many English boat owners limited the available maintenance and usage data. In addition, the English questionnaire was not well designed, including open questions with multiple elements. The French version was much improved, and all the boat owners were present to contribute.

The data are 14 – 17 years old. An increase in the average number of NIS per boat since then seems probable, mirroring increases seen in marina surveys [15]. (N.B. The hull fouling data could provide informative comparisons with new surveys.)

## Ethics Statement

The current work does not include animal experiments or data from social media platforms. Boat owners who were willing to participate were interviewed with the aid of a questionnaire to establish the specification of their vessel and its recent maintenance and usage history, exclusively factual information of low personal sensitivity centred on fouling of vessels. The boats and marinas involved in the study have been encoded in the data set, so original names are not included. Boat owner personal details were not relevant to the data set. No ethical approval was required.

## Credit Author Statement

**Christine Wood**: Data Curation, Methodology, Investigation, Writing - Review & Editing; **Frédérique Viard**: Conceptualization, Methodology, Formal analysis, Writing - Review & Editing, Supervision, Project administration, Funding acquisition; **Anna Yunnie**: Methodology, Investigation, Writing - Review & Editing; **Camille Bridge**: Methodology, Investigation, Data curation, Formal analysis; **John Bishop**: Conceptualization, Methodology, Formal analysis, Investigation, Writing - Original Draft, Visualization, Supervision, Project Administration, Funding acquisition.

## Data Availability

Mendeley DataFouling on recreational boats in the western English Channel (Original data).

## Appendix Supplementary materials

Boat hull fouling questionnaire Brittany Image, application 1

Boat hull fouling questionnaire Devon Image, application 2

Comparison of boat and marina pontoon survey data Image, application 3
