# Supplementary material for: Data on the extent of sessile invertebrate fouling on the hulls of recreational boats in the western English Channel (north-east Atlantic), and patterns of boat maintenance and usage there
Source: Data Brief. 2026 Apr 28;66:112803. doi: 10.1016/j.dib.2026.112803 (PMC13186057; doi:10.1016/j.dib.2026.112803)
Supplement: Boat hull fouling questionnaire Brittany [file mmc1.pdf]

SURVEY SITE :	DATE OF SURVEY :

This questionnaire aims to understand the sailing and maintenance history of your boat (to help us understand and comment on species and biodiversity records). The questionnaire is voluntary, anonymous and has no consequences for the owner.

BOAT DESCRIPTION

NAME	
LENGTH	
TYPE	☐ **Sailing :** ☐ Keel ☐ Centreboard ☐ Weighted centreboard<br>☐ **Motorboat**
HULL MATERIAL	☐ Fibreglass/GRP ☐ Wood ☐ Steel ☐ Aluminium
HOME PORT	

SAILING ACTIVITY

MAIN ACTIVITY	☐ Pleasure ☐ Fishing ☐ Racing
BERTH NO. or position	

DURING THE LAST SAILING SEASON	
**Longest period stationary at anchor (without going to sea e.g. winter)**	From : To :
**Type of mooring**	☐ Dock ☐ Buoy ☐ Mooring block ☐ Other :
**Was the boat taken out of the water? (outside of drying period for antifoul)**	☐ No<br>☐ Yes, when was the last time:<br>For how long:
**Have you sailed or anchored your boat in freshwater?**	☐ No<br>☐ Yes, when was the last time:<br>For how long:
**Have you practiced grounding /beaching?**	☐ No<br>☐ Yes, when was the last time:<br>For how long:
**Have you anchored on a mooring block?**	☐ No<br>☐ Yes

TYPE OF SAILING UNDERTAKEN DURING THE LAST SAILING SEASON (particularly since the last maintenance)				
				How many times ? e.g. once a year, once a month, once a week
**In the bay**	☐ for a day	☐ 2 to 3 days	☐ more	
**North Brittany**	☐ for a day	☐ 2 to 3 days	☐ more	
**Cross-Channel**	☐ for a day	☐ 2 to 3 days	☐ more	
**South Brittany**	☐ for a day	☐ 2 to 3 days	☐ more	
**Further away**	☐ for a day	☐ 2 to 3 days	☐ more	

Please check your destinations on the 'Sailing and Mooring Locations' form

HULL MAINTENANCE

**Date of last antifouling**	
**Who did the work?**	☐ Yourself ☐ A company
**Name of antifoulant used**	
**Type of antifoulant used**	☐ Hard matrix ☐ Semi-erodible ☐ Erodible
**Method of application**	☐ Roller ☐ Brush ☐ Spray-gun<br>☐ Other :
**Is antifoulant applied at each cleaning?**	☐ Yes ☐ No, frequency :

**Do you clean the hull between antifoul applications?**	
☐ **NO**	
☐ **YES** **IN WATER** ☐ Diving, fins-mask-snorkel ☐ Scuba diving ☐ Using a rib<br>**OUT OF WATER** ☐ Lifting or dry dock ☐ Grounding/beaching	
**Frequency of these cleanings :**	
**What is the reason?**	
**Date of last cleaning**<br>(date of last antifouling if no intermediate cleaning)	
