# Supplementary material for: Data on the extent of sessile invertebrate fouling on the hulls of recreational boats in the western English Channel (north-east Atlantic), and patterns of boat maintenance and usage there
Source: Data Brief. 2026 Apr 28;66:112803. doi: 10.1016/j.dib.2026.112803 (PMC13186057; doi:10.1016/j.dib.2026.112803)
Supplement: Boat hull fouling questionnaire Devon [file mmc2.pdf]

marine aliens

Hull Fouling Questionnaire

As part of a wider programme on marine alien species, we are looking at boat hulls to try to understand how marine creatures are moved around. We would be really grateful if you help us to complete this questionnaire about how you maintain and use your boat. It is completely voluntary and anonymous, neither your name or that of your boat will be used in any reports or publications.

Description and use of boat:

Name (or reference number):

Length:

Description/type:

Hull material (e.g. fibreglass, concrete, wood):

Sailing Activity:

- Predominantly cruising
- Predominantly racing
- Racing only
- Other

Berthing and voyages:

Where was the boat principally moored in the last 12 months? (Name of home marina or other mooring location):

When in the last 12 months was the boat moored at this site? (Specify months e.g. June to October):

Does this site partially or fully dry out at low tide?:

Was the boat taken out of the water last winter?:

Location and type of mooring e.g. berth number in marina or depth of water in bay, how far offshore, swinging or fixed mooring:

Longest stationary period in the water in last 12 months:

Where has the boat travelled to within the last 12 months? *(Use prompt list)*:

Does this include freshwater or brackish locations? If so, when was that?:

Hull Cleaning:

When did you last clean your hull in the last 12 months?:

- Was this cleaning in-water? If yes, was it by hand (using brush) or by SCUBA/ snorkelling? OR
- Was cleaning out of water? If yes, was it done by yourself or professionally?

- Are any areas usually missed (e.g. keel, rudder, logger?)

Was antifouling applied at the same time as cleaning? If not, when was antifouling last applied?:

What kind of anti-fouling paint was used? (Product name/manufacturer.) *(Use prompt list)*:

Method of application e.g. by yourself or professionally; brush, roller or spray:

Do you have any specific problems with hull fouling?:

Do you generally clean your hull between antifouling treatments? If so, what prompts this cleaning?:
