# Supplementary material for: Data on the extent of sessile invertebrate fouling on the hulls of recreational boats in the western English Channel (north-east Atlantic), and patterns of boat maintenance and usage there
Source: Data Brief. 2026 Apr 28;66:112803. doi: 10.1016/j.dib.2026.112803 (PMC13186057; doi:10.1016/j.dib.2026.112803)
Supplement: Comparison of boat and marina pontoon survey data [file mmc3.docx]

**Comparison of boat and marina pontoon survey data**

Occurrence (+) of sessile animal NIS at four marinas subjected to rapid assessment surveys (RASs) of the pontoons in 2010 and repeated in 2013, plus surveys of leisure craft at the same sites in 2008 - 2011 (QB, Devon) and in 2011 (MB, TB and AW, Brittany). *Botrylloides* spp. = *B*. *diegensis* plus *B. violaceus.* RAS data are from Bishop et al. (2015), and for the boats only the taxa reported in the RAS publication are shown.

| **Marina** | **QB** | | | **MB** | | | **TB** | | | **AW** | | |
| --- | --- | --- | --- | --- | --- | --- | --- | --- | --- | --- | --- | --- |
| **Survey** | Boats n = 71 | RAS  2010 | RAS  2013 | Boats  n = 21 | RAS 2010 | RAS 2013 | Boats  n = 18 | RAS 2010 | RAS 2013 | Boats  n = 10 | RAS  2010 | RAS  2013 |
| **Species** |  |  |  |  |  |  |  |  |  |  |  |  |
| *Didemnum vexillum* |  |  |  | **+** | **+** | **+** |  | **+** | **+** |  | **+** | **+** |
| *Perophora japonica* |  | **+** | **+** | **+** | **+** | **+** | **+** | **+** | **+** |  | **+** | **+** |
| *Corella eumyota* | **+** | **+** | **+** | **+** | **+** | **+** | **+** | **+** | **+** | **+** | **+** | **+** |
| *Styela clava* | **+** | **+** | **+** | **+** | **+** | **+** | **+** | **+** | **+** | **+** | **+** | **+** |
| *Asterocarpa humilis* | **+** |  | **+** | **+** | **+** | **+** | **+** | **+** | **+** | **+** | **+** | **+** |
| *Botrylloides* spp. | **+** | **+** | **+** | **+** | **+** | **+** | **+** | **+** | **+** | **+** | **+** | **+** |
| *Bugula neritina* | **+** | **+** | **+** | **+** | **+** | **+** | **+** | **+** | **+** | **+** | **+** | **+** |
| *Tricellaria inopinata* | **+** | **+** | **+** | **+** | **+** | **+** | **+** | **+** | **+** | **+** | **+** | **+** |
| *Watersipora subatra* | **+** |  | **+** | **+** | **+** | **+** | **+** | **+** |  |  | **+** | **+** |
| *Magallana gigas* |  |  | **+** | **+** | **+** | **+** |  |  |  |  |  |  |
| *Crepidula fornicata* | **+** |  | **+** |  |  |  |  | **+** |  |  |  |  |
| *Austrominius modestus* | **+** | **+** | **+** | **+** | **+** | **+** | **+** | **+** | **+** | **+** | **+** | **+** |
|  |  |  |  |  |  |  |  |  |  |  |  |  |
| **Total No. of NIS** | 9 | 7 | 11 | 11 | 11 | 11 | 9 | 11 | 9 | 7 | 10 | 10 |
| **RAS gain/loss 2010 - 2013** |  | +4 | |  | 0 | |  | -2 | |  | 0 | |

**REFERENCE**

J.D. Bishop, C.A. Wood, L. Lévêque, A.L. Yunnie, F. Viard, Repeated rapid assessment surveys reveal contrasting trends in occupancy of marinas by non-indigenous species on opposite sides of the western English Channel, Mar. Pollut. Bull. 95 (2) (2015) 699-706.
